# Exploring behavioral intervention components for African American/Black and Latino persons living with HIV with non-suppressed HIV viral load in the United States: a qualitative study

**DOI:** 10.1186/s12939-023-01836-3

**Published:** 2023-01-31

**Authors:** Sabrina R. Cluesman, Marya Gwadz, Robin Freeman, Linda M. Collins, Charles M. Cleland, Leo Wilton, Robert L. Hawkins, Noelle R. Leonard, Elizabeth Silverman, Carey B. Maslow, Khadija Israel, Amanda Ritchie, Sarah Ory

**Affiliations:** 1 Intervention Innovations Team Lab (IIT-Lab), New York University Silver School of Social Work, 1 Washington Square North, Room 303, New York, NY 10003 USA; 2 Center for Drug Use and HIV Research, School of Global Public Health, New York University, New York, NY USA; 3 Independent Consultant, Brooklyn, NY USA; 4 Department of Social and Behavioral Sciences, School of Global Public Health, New York University, New York, NY USA; 5 Department of Population Health, Division of Biostatistics, New York University School of Medicine, New York, NY USA; 6 Department of Human Development, State University of New York at Binghamton, Binghamton, NY USA; 7 Faculty of Humanities, University of Johannesburg, Johannesburg, South Africa; 8 College of Humanities and Social Sciences, North CarolinaState University, Raleigh, NC USA; 9 School of Global Public Health, New York University, New York, NY USA; 10 Independent Consultant, New York, NY USA

**Keywords:** Qualitative, HIV care continuum, Racial/ethnic inequities, Intervention components, Optimization trial, Multiphase optimization strategy (MOST), Critical race theory, Harm reduction, Self-determination theory, Structural salience, Cultural salience

## Abstract

**Background:**

The persistence of racial/ethnic inequities in rates of engagement along the HIV care continuum signals the need for novel approaches. We developed six behavioral intervention components for use in an optimization trial, grounded in a model that integrates critical race theory, harm reduction, and self-determination theory, designed to address various barriers that African American/Black and Latino persons living with HIV (PLWH) experience to the HIV care continuum. The components were: health education, motivational interviewing sessions, pre-adherence skill building, peer mentorship, focused support groups, and navigation. The present qualitative exploratory study describes participants’ perspectives on the components’ acceptability, feasibility, and impact.

**Methods:**

Participants were African American/Black and Latino PLWH poorly engaged in HIV care and with non-suppressed HIV viral load in New York City. From a larger trial, we randomly selected 46 participants for in-depth semi-structured interviews. Interviews were audio-recorded and transcribed verbatim, and data were analyzed using directed content analysis. Quantitative data on sociodemographic and background characteristics and components’ acceptability and feasibility were also collected.

**Results:**

On average, participants were 49 years old and had lived with HIV for 19 years. Most were cisgender-male and African American/Black. Participants reported a constellation of serious social and structural challenges to HIV management including chronic poverty, unstable housing, and stigma. Across components, a non-judgmental and pressure-free approach and attention to structural and cultural factors were seen as vital to high levels of engagement, but lacking in most medical/social service settings. Prominent aspects of individual components included establishing trust (health education); developing intrinsic motivation, goals, and self-reflection (motivational interviewing sessions); learning/practicing adherence strategies and habits (pre-adherence skill building); reducing social isolation via peer role models (peer mentorship); reflecting on salient goals and common challenges with peers without stigma (focused support groups); and circumventing structural barriers to HIV management with support (navigation). Components were found acceptable and feasible. Findings suggested ways components could be improved.

**Conclusions:**

The present study advances research on interventions for African American/Black and Latino PLWH, who experience complex barriers to engagement along the HIV care continuum. Future study of the components is warranted to address racial/ethnic health inequities in HIV.

**Supplementary Information:**

The online version contains supplementary material available at 10.1186/s12939-023-01836-3.

## Introduction

African American/Black and Latino (AABL) persons living with HIV (PLWH) evidence substantial and long-standing inequities in rates of engagement along the HIV care continuum (namely, the necessary steps in HIV management including linkage to and retention in HIV care, uptake of HIV medication, and HIV viral suppression) [1, 2]. The severity and persistence of these inequities along the HIV care continuum signal the need for innovations in conceptual models, behavioral intervention strategies, and intervention science methods [1, 2]. In particular, periods of non-suppressed HIV viral load have potential serious adverse effects on the quality of life and physical and mental health of PLWH [3]. Moreover, those with non-suppressed HIV viral load can potentially transmit HIV to others, while that risk is almost non-existent for those with sustained HIV viral suppression [4]. Poor engagement along the HIV care continuum among AABL PLWH results from a confluence of serious barriers and impediments, mainly at structural and social levels of influence [5–8]. Primary among these barriers are extreme and chronic poverty, unstable or sub-standard housing, unemployment, food insecurity, difficulties accessing high-quality care and services, social isolation, discrimination, and stigma [5–8]. Clearly, sustaining HIV viral suppression is challenging in this context [8]. Our research team focuses on identifying and understanding the complex and multi-level factors that drive poor engagement along the HIV care continuum among AABL PLWH and creating behavioral interventions to reduce racial/ethnic inequities in engagement [8–12]. Indeed, the larger public health goal of ending the HIV epidemic cannot be reached without increasing rates of sustained HIV viral suppression among PLWH, including AABL PLWH, and effective and efficient behavioral interventions have a vital role to play in this effort [13].

In a recent study, we described a new conceptual model developed by our research team, referred to as the Intervention Innovations Team integrated conceptual model (IIT-ICM), which combines critical race theory, the harm reduction perspective, and self-determination theory [2]. It was developed in response to a growing consensus in the field that many attempts to improve HIV care continuum engagement have insufficiently represented the underlying factors that drive poor engagement, including structural and cultural factors [14]. As we describe in more detail in that study, the three theories/approaches combine synergistically to produce a useful model. We used the IIT-ICM to design six behavioral intervention components to address a set of multi-level barriers to engagement along the HIV care continuum among AABL PLWH. The components are health education; motivational interviewing sessions; pre-adherence skill building; peer mentorship; focused support groups; and navigation. We describe the components and the theoretical barrier or barriers each seeks to address in more detail below.

These behavioral intervention components were tested with a study population of AABL PLWH who were not engaged in HIV care at recommended levels and who did not evidence HIV viral suppression at the time of enrollment [15]. To empirically test the components, we carried out an optimization trial grounded in the multiphase optimization strategy (MOST). In brief, the MOST framework is an engineering-inspired approach to examining the effects of individual intervention components, the most promising of which can then be combined into a multi-component intervention, and inactive or poorly performing components can be eliminated. The MOST framework and this optimization trial are described elsewhere [15, 16]. The present qualitative study seeks to understand the acceptability, feasibility, and impact of the behavioral intervention components from the participants' perspectives. These findings will be used in a number of ways: They will inform the next steps in the optimization trial (namely, adding valuable contextual information to the decision-making process about which components might be included in a multi-component intervention), our future research with AABL PLWH and research conducted by others, and the field of intervention science generally.

Acceptability and feasibility are the foundations of engagement in an intervention, with engagement defined as the depth of involvement with the behavior change process [17, 18]. In turn, engagement activates an intervention’s mechanisms of action, which then produce its effects on behavior [17]. We define intervention acceptability as how well an intervention is received by the target population, the extent to which the intervention might meet the needs of the target population, and, therefore, how satisfied participants are with it [18, 19]. Thus, acceptability is an experiential and affective experience of an intervention [17]. Study feasibility is defined as the proportion of participants attending assigned interventions, or usage [18]. Thus, feasibility is the behavioral aspect of engagement in an intervention [17]. The barriers that impede engagement along the HIV care continuum for AABL PLWH (e.g., poverty, distrust, stigma) also impede their participation in HIV research [10, 20], underscoring the importance of high-quality, acceptable, and feasible interventions, to boost engagement and potential effects. Further, to be effective, interventions must address a critical set of the population’s specific barriers to a health outcome [2]. We maintain that since interventions evolve through any given multi-stage program of research, there is utility to examining acceptability and feasibility through all research stages, not only in a program’s initial stages [21–23]. Thus, the present study explores the intervention components’ acceptability and feasibility.

### Conceptual model underlying the intervention components

The IIT-ICM was motivated in part by a research literature indicating existing behavioral interventions for AABL PLWH commonly lack sufficient structural and cultural competency and structural and cultural salience to be effective in this population [2, 14, 24, 25]. Structural competence is defined as the trained ability to discern how issues defined clinically as symptoms, attitudes, or diseases, such as medication “non-compliance,” depression, or smoking, represent the downstream implications of a number of powerful upstream influences such as health care delivery systems, zoning laws, and urban and rural infrastructures [25]. Cultural competency in health care describes the ability of systems to provide care to clients with diverse values, beliefs, and behaviors, including the tailoring of health care delivery to meet patients' social, cultural, and linguistic needs [26]. Lacking structural competency, interventions and staff in past studies may have failed to recognize ways in which clients’ reported symptoms, attitudes, or diseases may in fact be manifestations of structural factors (e.g., health care delivery systems, housing policy, transportation infrastructure, and federal entitlement benefit levels) that shape health and illness [25]. Lacking cultural competency, interventions and staff may have been insufficiently able to care for clients with diverse values, beliefs, and/or behaviors, and less able, or even unable, to tailor delivery of care that meets clients’ social, cultural, and linguistic needs [26]. The IIT-ICM is rooted in the understanding that to be effective, an intervention to reduce racial/ethnic inequities requires structural and cultural competency of staff, and structural and cultural salience of intervention content [25, 27]. We briefly describe relevant aspects of the IIT-ICM in the next section.

Critical race theory is one of the three pillars of the IIT-ICM and underscores the pervasiveness of institutional and systemic racism, including current and past abuses of AABL populations by medical research and medical institutions, and the resultant fear and distrust of medical settings and medications, counter-narratives, stigma, and structural challenges accessing health services [10]. Critical race theory emphasizes the value of focusing on the reality of lives lived within the context of normative, systemic racism (called ‘centering the margins’) and experiences occurring as a result, rather than focusing on race itself as a determinant of differential outcomes [28]. The second pillar of the IIT-ICM is the harm reduction perspective. Harm reduction ensures a non-judgmental, non-coercive approach to any positive change in health and related behaviors, including substance use, HIV medication adherence, and other domains in which harm to PLWH may be reduced [29]. Self-determination theory is the third pillar of the IIT-ICM to ensure attention is paid to the psychosocial experiences essential to developing, maintaining, and/or boosting durable intrinsic motivation to make behavioral changes. Self-determination theory conceptualizes human motivation as a product of three innate, fundamental psychological needs and the conditions in the larger environment that fosters them: autonomy (feeling in control of one’s own behaviors and goals), competence (being able to master tasks and learn different skills), and connection or relatedness (experiencing a sense of belonging and attachment to other people) [30, 31]. In practice, the IIT-ICM emphasizes the importance of staff being worthy of trust and building trust, a destigmatizing, non-judgmental, and dignity-enhancing approach, and seeking to guide participants toward any positive change they wish to make. The model further highlights the need for individualized care and autonomy-supportive practices using evidence-based techniques, and the importance of cultural and structural salience as noted above. As an integrated theory, we assume the three pillars have equal importance [2]. One aspect of the proposed study was to explore what core messages and key characteristics consistent with the ICM were “received” by participants if any. In other words, components were designed to address certain barriers and provide a certain type of clinical experience, but only the participants in the trial could tell us whether these core messages and key characteristics were indeed communicated and if they were useful.

#### Motivational interviewing

We identified motivational interviewing as a recommended counseling approach for use in interventions grounded in the IIT-ICM. Motivational interviewing seeks to nurture durable intrinsic motivation and readiness for behavior change by eliciting participants’ values, perspectives, and questions, identifying ambivalence and discrepancies, and, with permission, correcting misunderstood or inaccurate information [32, 33]. Self-determination theory is an accepted theoretical underpinning of motivational interviewing [32] and motivational interviewing is therefore consistent with the core elements of the IIT-ICM. Effective at clinically significant levels when applied to a range of health behaviors [34–36], motivational interviewing has proven especially effective among AABL, compared to White populations [34]. Motivational interviewing may be of particular use in contexts where self-determination is *not* often fostered and as a result, durable intrinsic motivation for change may be lacking [10–12]. The behavioral intervention components were grounded in the general motivational interviewing approach and also drew on other behavior change techniques as appropriate, as described below.

In summary, each behavioral intervention component had several features. Each was grounded in the IIT-ICM, which can be thought of as the component’s underlying ethos. Second, each intervention component had a modality; that is, how the component was delivered (e.g., individually, in groups, with peer mentors). Third, intervention components had specific content, structured in the form of interactive activities, informed by existing evidence-based interventions and techniques (e.g., needs assessment to guide navigation, short videos to frame a discussion about barriers to health behavior, meetings with a peer mentor). Further, each component had an approach to behavior change. As noted above, we consider the motivational interviewing approach as naturally aligned with the IIT-ICM and essential to the IIT-ICM. Components included other counseling approaches congruent with motivational interviewing as needed, such as group dynamics theory. Within this approach or these approaches to behavior change, the intervention components also drew on other behavior change techniques appropriate for its overall goals. A behavior change technique is defined as a theory-based and systematic procedure included as an active component of an intervention designed to change behavior. Behavior change techniques are characterized as being observable, replicable, irreducible, and a postulated active ingredient within the intervention [37, 38]. Examples of behavior change techniques include habit formation, goal setting, and social support. Taken together, these elements that comprise a behavioral intervention component are intended to yield high levels of acceptability, feasibility, and engagement, and influence a particular mediator or small set of mediators, to thereby bring about behavior change.

The present qualitative study seeks to advance the understanding of behavioral intervention approaches for AABL PLWH who are poorly engaged along the HIV care continuum and who do not evidence HIV viral suppression. We describe the six behavioral intervention components in detail and explore participants’ perspectives on their acceptability, feasibility, mechanisms of action (whether related to the IIT-ICM or otherwise), ways the component may have influenced the participants’ attitudes, emotions, social relationships, and/or behavior, and ways the intervention components could be improved.

## Methods

### Description of the larger study from which qualitative data were drawn

The present study draws on qualitative data from a larger study, an intervention optimization trial that used an efficient fractional factorial design to test the effects of intervention components on a primary outcome, HIV viral suppression. In past research, we described this optimization trial, which was undertaken in New York City between 2014 and 2021, and which had the field name “Heart to Heart 2” [15]. A total of 512 participants were enrolled in the trial. Eligibility criteria were: 18–65 years of age; African American or Black and/or Latino or Hispanic ethnicity; diagnosed with HIV >  = 6 months ago; had taken < 50% of prescribed HIV medication during the past 6 weeks and had a non-suppressed HIV viral load level on a test carried out by a commercial lab; had < 1 HIV care visit in each 4-month period of the past year or missed > 2 visits without having canceled in the past year (pro-rated for those diagnosed less than a year ago); were residents of the New York City metropolitan area; were able to conduct research activities in English or Spanish; were willing to provide a blood specimen to assess HIV viral load at screening; and were willing to be randomly assigned to an experimental condition.

Participants were randomly assigned to one of 16 experimental conditions, each of which was comprised of a unique combination of intervention components. Most experimental conditions included 2–4 intervention components. Participants were enrolled in the optimization trial for approximately 12 months, and engaged in intervention activities for 4–8 months, depending on the experimental condition to which they were assigned. Participants were assessed with a structured measurement battery at baseline and 4-, 8-, and 12-months post-baseline. Assessments of HIV viral load were obtained from a commercial laboratory at screening and 8- and 12-months post-baseline. (Financial constraints prohibited obtaining viral load results at the 4-month follow-up.) Qualitative interviews were administered to a subsample of participants enrolled in the larger trial (N = 46). (More details on how participants were sampled for qualitative interviews are provided below.) Participants were compensated for all study activities (generally $25 for each activity). Participants gave signed consent for all study activities. Funding for local, round-trip public transportation was provided for study activities. Activities took place in a confidential setting at a project field site in lower Manhattan in New York City prior to the implementation of COVID-19 restrictions on in-person activities, after which activities were conducted over the telephone [39]. Thus, the optimization trial was implemented with a sample of AABL PLWH who at the time of enrollment did not engage in HIV care at recommended levels and who showed evidence of both poor adherence to HIV medication and non-suppressed HIV viral load [15, 16]. The optimization trial was approved by the Institutional Review Board at the New York University Grossman School of Medicine. The optimization trial and its design elements are described in more detail elsewhere [15].

Table 1 presents the six behavioral intervention components to ameliorate barriers to HIV care continuum engagement for AABL PLWH. Each intervention component was designed to address and ameliorate a specific mediator or small set of theoretical mediators. In Table 1, the theoretical mediators that each component was designed to address are shown, as are the components’ goals, modality, behavior change techniques, content source (if any), and select examples of how the component was designed to be culturally and/or structurally salient, consistent with the IIT-ICM. Intervention component implementation was guided by manuals structured as a sequence of interactive exercises and activities, but also were flexible with options for individualized material based on participants' own concerns and circumstances. Intervention components were administered by clinically trained, culturally and structurally competent master’s level interventionists trained in motivational interviewing techniques and the behavior change techniques used in the specific component (e.g., group counseling). Within the context of a general presumption that HIV medications are participants’ best chance for a longer and higher quality of life, interventionists elicited and respected participants’ personal perspectives and decisions about HIV medication including the decision to decline HIV medication. Participants were referred to their health care providers for individual medical recommendations. In addition to Table 1, we briefly describe the structure and content of each intervention component in the Results section, for clarity and ease of interpretation of study findings. Regarding the order in which components were administered, the core session (health education) was provided first to all participants. (An optimization trial typically provides a core session to all participants.) Then, the navigation component began (either 3- or 6-months in duration) so that structural barriers to HIV care engagement could be addressed at the outset of the intervention period. The motivational interviewing sessions component was the second component administered for those participants randomly assigned to an experimental condition that provided it, followed by the peer mentorship and focused support groups components, which could be administered simultaneously, although not on the same day. Pre-adherence skill building was the last component administered for those assigned to an experimental condition that included that component.

**Table 1:** Description of the intervention components explored in the present study

**Name**	**Theoretical mediators**	**Description, specific behavior change techniques, and relationship to the integrated underlying conceptual model**
All components	–	▪ Guided by the IIT-ICM and uses the motivational interviewing approach, in addition to evidence-based approaches and behavior change techniques specific to that component ▪ Constructed to address or circumvent the primary multi-level barriers that AABL PLWH experience to HIV care continuum engagement and to be both culturally and structurally salient ▪ Takes the stance that HIV medication is PLWH’s best chance for a long and healthy life and optimal quality of life at the population level, but does not presume HIV medication is “right” for any individual participant at this time; elicits and respects personal decisions about HIV care and medication ▪ Considers substance use as a barrier ▪ Guided by manuals comprised of interactive exercises; manuals are flexible, individualized (i.e., elicits and attends to barriers related to race/ethnicity, social class, sex, time living with HIV, and sexual/gender minority status) ▪ Led by a clinically trained master’s level interventionist with expertise in commitment to structural and cultural competency and motivational interviewing ▪ Interventionists do not make individual medical recommendations but instead refer participants back to their health care providers; staff members’ level of medical knowledge is comparable to a health educator
Core session	HIV health education	▪ All participants receive the core session ▪ **Goals**: Foster engagement, build trust and relationships between the project and participant, increase or reinforce fundamental knowledge of recommended HIV care engagement and HIV medication use ▪ **Modality**: 1 health education session held in-person with participants individually, < 60 min ▪ **Approach/Behavior change technique**: Health education ▪ **Content:** Standard treatment education on the current U.S. Department of Health and Human Services recommendations for frequency of HIV care appointments, timing of HIV medication initiation, and importance of high levels of adherence ▪ **Examples of cultural/structural salience:** Implicitly recognizes that not all AABL PLWH attend HIV care appointments and take HIV medication due to complex multi-level barriers (structural salience, trust building, de-stigmatizing, individualized care); presumes participants are experts on their own health but that health education may be welcome (strengths-based, dignity enhancing)
A. Motivational Interviewing Sessions	Health beliefs (e.g., outcome expectancies, self-efficacy); and emotions (e.g., medical distrust, fear of HIV medication)	▪ **Goals:** Guide participants in uncovering and articulating ambivalence about and exploring and resolving challenges to behavior change to thereby foster movement toward personal health goals (HIV-related or otherwise) ▪ **Modality**: 4 in-person sessions held with participants individually, approximately 60 min each ▪ **Approach/Behavior change techniques:** Uses specific motivational interviewing techniques such as articulating emotions and values, identifying discrepancy, setting personal goals and barriers to achieving goals, problem-solving barriers, planning ▪ **Content:** Each session includes 1–2 culturally and structurally salient video narrative segment(s) to highlight key issues (e.g., distrust, fear, counter-narratives, poverty, stigma) and foster discussion about these topics ▪ Session 1 addresses barriers to HIV care; Sessions 2 and 3 target barriers to HIV medication (S2: evoking barriers, fostering readiness; S3: decisions, plans); Session 4 addresses medication adherence barriers and their solutions in depth and finalizing care/HIV medication plans ▪ **Examples of cultural/structural salience**: Explicit focus on specific barriers to care/medication that are prevalent among AABL PLWH such as medical distrust, fear, counter-narratives (trust building, culturally salient, structurally salient), incorporates participants’ own views on how to best manage HIV and medication without judgment (non-judgment, individualized care, autonomy support)
B. Pre-Adherence Skill Building	Behavioral skill to manage HIV medication adherence (habits)	▪ **Goals**: Prepare the physical and social “adherence environment,” put long-term HIV medication supports in place, and build adherence skills including using pill boxes, visual aids and reminders, and building adherence habits ▪ **Modality:** Six-week intervention period with meetings between the interventionist and participant at the beginning and end of the intervention period, approximately 60 min each, and brief weekly check-in phone calls. Thus, conducted with participants individually in-person (session 1 and 6) and by phone (sessions 2–5) ▪ **Approach/Behavior change techniques:** Identify barriers to and facilitators of HIV medication adherence in the physical and social environments, action planning, learn and practice habit formation (behavioral practice), and feedback ▪ **Content**: Guided by the US Health Resources and Services Administration (HRSA) guidelines for preparing PLWH for treatment success ▪ Those who do not wish to take medication at this time can apply the concepts to other health behaviors ▪ First session assesses readiness for medication, identifies individual barriers to adherence prior to initiating medication (e.g., lack of supports, active substance use), links adherence to daily activities to build habits (e.g., taking medication at the same time as another regular activity), puts educational and visual aids and reminders in place (e.g., pill boxes, alarms), identifies long-term supports/supporters who can reinforce successes, and plan to minimize lapses if doses are missed ▪ Participants then have the opportunity to practice and build adherence habits. The interventionist will check in with the participant weekly to discuss and provide feedback. Barriers of/facilitators to adherence, if any, will be explored ▪ Final session to review progress made and future plans ▪ **Examples of cultural/structural salience:** Reflects the major practical and psychosocial barriers AABL PLWH experience to viral suppression such as lack of privacy in home setting, stigma, fear (structurally salient)
C. Peer Mentorship	Peer modeling and peer norms (primary), social support and stigma (secondary)	▪ **Goals:** Provide credible “successful” role models and challenge negative peer norms about HIV care engagement and HIV medication use, provide social support and reduce stigma ▪ **Modality:** Four-month intervention period with individual meetings held approximately weekly in-person or by phone (including two-way text messaging communication). The initial meeting is held in-person ▪ **Approach/Behavior change techniques:** Modeling of behavior, social support (practical and emotional), feedback on behavior, social comparison with peer mentor ▪ **Content:** Based on the HRSA-funded Peer Education & Evaluation Resource (PEER) model ▪ Participants are linked with a “successful” peer mentor (i.e., a demographically similar PLWH who has consistently engaged in HIV care and is taking HIV medication with high levels of adherence) ▪ The role of the peer mentor is to: model healthy HIV behavior; provide practical tips for managing HIV care/medication based on their personal experience; and provide social support. The peer’s experience managing HIV and willingness to share personal experiences reduces stigma and challenges social norms that AABL PLWH are typically poorly engaged along the HIV care continuum ▪ **Examples of cultural/structural salience:** Peer is also AABL and living with HIV and an expert in the barriers this population experiences to engagement along the HIV care continuum, as well as solutions to barriers (strengths-based, trust building, de-stigmatizing)
D. Focused Support Groups	Social support and stigma regarding care/ HIV medication status	▪ **Goals:** Provide emotional and instrumental social support for health goals and other concerns, reduce stigma ▪ **Modality:** 6 in person groups, ~ 90 min, every 2–3 weeks, over 4 months ▪ **Approach/Behavior change techniques**: Social comparison, demonstration of the behavior, and social support, and encourage shifts in perspective in a group process context ▪ **Content**: Group members choose a primary topic to discuss ▪ Topics include barriers to and decisions regarding care/HIV medication, reasons for not taking HIV medication, distrust and fear of HIV medication, race/ethnicity and social class and HIV medication, substance use and HIV medication, coping with HIV medication, managing pressure to take HIV medication in HIV care, mental health, stigma, managing communication with health care providers, and adherence to HIV medication ▪ **Examples of cultural/structural salience:** Groups addressed major barriers AABL PLWH experience to engagement along the HIV care continuum that are not commonly discussed in other settings such as medical distrust, fear of HIV medications, counter-narratives, substance use issues (de-stigmatizing, culturally salient, structurally salient)
E. Navigation	Ameliorating structural barriers to care and HIV medication	▪ Participants receive either the short (three month) or long (six month) duration of this component ▪ **Goals**: Identify needs and barriers to meeting needs and address or circumvent structural barriers to HIV care and ancillary services in the context of poverty ▪ **Modality**: Weekly sessions held with participants individually, initial meeting in person (< 90 min) and then weekly meetings in-person or via phone. Menu-based and highly focused ▪ **Approach/Behavior change techniques:** Needs assessment to assist PLWH in identifying barriers to health services for HIV and other needs (e.g., for substance use and mental health, housing, insurance), problem-solving and navigation to circumvent or resolve barriers ▪ **Content**: Based on the HRSA HIV System Navigation model. Navigation is an efficacious, flexible, individualized, strengths-based approach ▪ Comprised of an initial face-to-face meeting (30–60 min.) for review of participant’s readiness for and barriers to care/HIV medication, including substance use and mental health, and creation of a Change Plan/Action Plan ▪ A minimum of weekly phone (including text messages), email, and in-person meetings during the navigation period, depending on need ▪ The menu of activities includes screening and “Fast Track” referrals for housing, substance use, mental health, and other concerns; problem-solving barriers to appointments; and accompaniment to health care appointments ▪ **Examples of cultural/structural salience**: Designed to address the primary structural barriers that AABL PLWH experience to HIV care, HIV medication, and ancillary services such as for substance use, mental health (individualized care, structurally salient)

### Design of the present study

The qualitative interviews carried out as part of the optimization trial were designed to elicit participants’ perspectives on the acceptability, feasibility, mechanisms of action, and impact of the specific behavioral intervention components, along with suggestions for improvement. The qualitative sample was comprised of 2–4 randomly sampled participants from each of the 16 experimental conditions tested in the optimization trial. Although random sampling is not the predominant sampling method for qualitative research (e.g., in contrast to a method such as purposive sampling for maximum variability), we chose random sampling to ensure that participants from each experimental condition were included in the qualitative subset. Moreover, random sampling within experimental conditions had a similar goal of capturing maximum variability in participant characteristics. For each participant selected, two in-depth qualitative interviews were scheduled, the first within 3–5 months of enrollment in the optimization trial (and toward the beginning of the participant’s intervention period); the second upon completion of the intervention period. A total of 46 participants engaged in the first qualitative interview and 32 of these also completed the second interview (thus 70% participated in both interviews). Participants received $25 for each qualitative interview. Qualitative interviews were audio-recorded and professionally transcribed verbatim. The qualitative interviews with 46 participants enrolled in the larger optimization trial comprise the data set for the present study. The present study also draws on quantitative data from the optimization trial to describe participants’ socio-demographic, background, and health characteristics, intervention acceptability ratings, and feasibility rates. Although we present some quantitative data, we consider the present paper to be primarily a qualitative exploration and not rise to the level of a mixed-methods study.

### Qualitative measure

Qualitative interviews were administered to participants by trained interviewers using a semi-structured guide developed by the research team, which included experts on AABL PLWH and the HIV care continuum, and which was rooted in the IIT-ICM and a review of relevant literature. Structured as a series of suggested questions and prompts, the guide directed the interviewer from general to more specific questions in each of four sections: 1) overall experiences with the study (e.g., To start off, what was it that led you to agree to participate in the study? What stands out to you most about the project so far? What have you liked? Disliked? Have you been involved in similar projects? How is this study similar/different? What do you think about the staff in general?); 2) emotional or behavioral effects of study participation or recent changes concurrent with study participation, if any (e.g., Have you taken HIV medications since you joined the Heart to Heart 2 study? It’s OK if you haven’t. We just want to understand what’s going on with you now. Why or why not?, What factors played a role in your deciding to take HIV medications at this time, whether related to the Heart to Heart 2 study or other factors?, Since you’ve been involved with the study, has anything changed about the way you think about HIV medication?); 3) exploration of the specific intervention components the participant was randomly assigned to receive (e.g., What do you remember about this component?, Can you tell me what kinds of things you discussed? Describe whether this component useful to you or not useful?); and 4) ways the study could be improved (e.g., We want to ask a few more questions about how we can improve. What do you think should be included in the Heart to Heart 2 study that wasn’t included?). Pilot-tested and refined prior to being used, the guide was updated periodically to incorporate emergent constructs (e.g., the experience of being pressured to take HIV medication and its effects). The interview guide is provided as supplemental material. (Note that the present study uses a subset of the qualitative data collected and therefore the complete set of questions included in the guide is not summarized above.)

### Qualitative analyses

Analyses of qualitative data followed a directed content analysis approach that was both inductive and theory-driven [40]. We started with an initial list of “start codes” and their operational definitions that was generated by the primary qualitative analyst, a medical anthropologist. This initial start code list was informed by the theories and perspectives framing the study including the IIT-ICM. Codes were generated that reflected known salient factors such as structural barriers (e.g., quality of housing, poverty), culture and race/ethnicity (e.g., experiences of discrimination, medical distrust, counter-narratives); substance use management; autonomy, competence, and relatedness; and other factors known to promote or impede engagement along the HIV care continuum (e.g., mental health distress). Using this scheme, the primary analyst coded interview transcripts along with an additional trained qualitative researcher. During the coding process, codes were refined, clarified, and/or broadened; for example, when new codes were identified. Discrepancies in codes and coding between the data analysts were resolved by consensus. Then, the interview transcripts were recoded using the final coding frame. Further, a subset of transcripts were coded using the final coding frame by three other members of the research team. Codes were then combined into larger themes and sub-themes in an iterative process led by the two main data analysts and in collaboration with an interpretive community of research team members, which included cisgender men and women, people who identified as transgender, gender non-binary, or gender-fluid, people from White, African American/Black, Asian, and Latino/a backgrounds, and PLWH [41, 42].

Methodological rigor of the analysis was monitored continually in several ways. An audit trail of the process and analytic memos was maintained [43]. Analysts engaged in periodic debriefing sessions with the interpretive community. The primary analysts and the interpretive community attended to the potential effects of the team’s positionality related to power and privilege, sex, gender, race/ethnicity, health, and socioeconomic status throughout the data collection process through reflection and training that focused on how these factors might affect interviewing and data analytic processes [44, 45]. In addition, member-checking was conducted with AABL PLWH, and feedback from member-checking was incorporated into the results [43]. Although we used the random sampling method for the qualitative interview as described above, we attended to issues of maximum variation in sample characteristics [46] as one aspect of trustworthiness [47].

### Quantitative measures

Structured instruments developed specifically for HIV populations in high-risk contexts were used to assess relevant quantitative domains, including age, sex assigned at birth, gender identity, sexual minority status (identifies as gay, lesbian, bisexual, queer, or other non-heterosexual), race/ethnicity, housing status, history of incarceration, and indicators of extreme poverty (how often unable to pay for necessities in the past year, and how often experienced food insecurity in the past year) [48]. Years since first HIV diagnosis, years since first initiated antiretroviral therapy, and months since last HIV medication dose (if not on HIV medication at screening) were assessed using a version of the HIV Cost and Services Utilization Study instrument (HCSUS) [49]. HIV viral load level and HIV viral suppression (< 200 copies/mL) were assessed by laboratory report. The number of adverse experiences in early life, including peer victimization, neighborhood disorder, physical abuse, sexual abuse, and neglect, were assessed using the 14-item Adverse Childhood Experiences Scale-Revised (ACES-R) [50]. Likely depression was determined using established thresholds on the Patient Health Questionnaire (PHQ-9) depression module [51]; likely anxiety was determined using the Generalized Anxiety Disorder Scale (GAD-7) [52], and likely post-traumatic stress disorder (PTSD) was assessed with the Primary Care PTSD Screen [53]. Patterns of substance use were assessed using the World Health Organization Alcohol, Smoking and Substance Involvement Screening Test (WHO ASSIST) which provides scoring algorithms to distinguish substance use at moderate-to-high risk vs. low-risk levels [54]. The Client Satisfaction Survey [55] was used to determine intervention component acceptability (data were recoded to indicate the proportion of those who attended the component who found it ‘quite a bit helpful’ to ‘very helpful’). A domain was considered acceptable if ≥ 70% found it ‘quite a bit helpful’ to ‘very helpful.’” Study feasibility was defined as the proportion of participants attending assigned components. A component was feasible if ≥ 70% attended.

### Quantitative analyses

Descriptive statistics were used to summarize quantitative measures to describe the socio-demographic and background composition of the qualitative study sample and to summarize measures of study acceptability and feasibility from the larger study sample.

## Results

### Sample description

Table 2 presents sociodemographic and background characteristics describing participants in the present study. The average age was 49 years, and a large majority (78%) were assigned male sex at birth. One-third (33%) identified as sexual and/or gender minorities. Most (76%) were African American or Black, and over half (52%) were not in stable housing. The average number of adverse childhood experiences was approximately 4 (SD = 3, range:0–14) and 50% had an ACES-R score of 4 or higher. Only 17% of participants were employed and, in the past year, nearly half (46%) had insufficient funds for necessities at least monthly, and most (85%) had sometimes or often experienced food insecurity. Nearly one-fifth (17%) had engaged in transactional sex during the past year. The average time since HIV diagnosis was 19 years (SD = 7 years, range:3–30 years). Thus, participants, on average, were long-term HIV survivors. All participants had taken HIV medication in the past, with the longest duration of sustained use being 45 months (SD = 63, range: 0–264). A moderate-to-high risk level of alcohol use was reported by approximately half of participants (54%); for cannabis use, this proportion was 61%, and for cocaine or crack use it was 63%. Most (78%) had engaged in treatment for substance use in the past. Thus, substance use at potentially hazardous levels was common in this sample. Most (87%) had never injected drugs; among those who had, half had done so in the past 3 months. Likely depression, anxiety, or PTSD was reported by 22%, 11%, and 35%, respectively. Two-fifths (40%) of participants showed evidence of suppressed HIV viral load at the 8- and/or 12-month follow-up assessment (we did not assess HIV viral load at the 4-month assessment).

**Table 2:** Participant sociodemographic and background characteristics (*N* = 46)

	**M (SD) or %**
Age (range 23 – 62 years)	48.9 (8.74)
*Sex assigned at birth*	
Female	21.7
Male	78.3
Sexual and/or gender minority status	32.6
Transgender gender identity, gender fluid, gender non-conforming	4.3
African American or Black (non-Latino/Hispanic)	76.1
Latino or Hispanic	21.7
Stable housing (has their own home or apartment, including funded by government programs or benefits)	47.8
Adverse Childhood Experiences (ACES-R) score (range 0–14)	3.56 (3.33)
ACES-R score = 4 or higher	50.0
*Indications of low socioeconomic status and extreme poverty*	
Working full-time or part-time off-the-books or on-the-books	17.4
Ran out of funds for necessities monthly or more in the past year	45.7
Food insecurity often or sometimes in past year	84.8
Engaged in transactional sex – past year	17.4
*HIV-related factors*	
Years since HIV diagnosis at enrollment (range 3.0—30.0 years)	18.6 (7.18)
Median [Q1, Q3]	18.5 [13.3, 24.0]
Took HIV medication in the past	100
Times stopped/started HIV medication in the past (range 0–100)	10.6 (17.8)
Longest duration of sustained HIV medication, in months (range 0–264)	45.0 (62.7)
*Psychosocial risk and protective factors*	
Alcohol use at a moderate-to-high-risk level	54.3
Cannabis use at a moderate-to-high-risk level	60.9
Cocaine or crack use at a moderate-to-high-risk level	63.0
Use of other drugs (not including alcohol, cannabis, cocaine/crack) at a moderate-to-high-risk level	28.3
Never injected drugs	87.0
Injection drug use lifetime, but not in the past 3 months	6.5
Injection drug use – past 3 months	6.5
Participated in substance use treatment in the past	78.3
Likely depression	21.7
Likely anxiety	10.9
Likely PTSD	34.8
*HIV viral load*	
HIV viral load level at enrollment (log_10_ transformed)	4.28 (0.970)
Suppressed HIV viral load at 8- and/or 12- month follow-up assessment	40.0

## Overview of results

Overall, participants reported their engagement with the intervention components provided a unique physical and emotional space — which they noted was not commonly found in the other social service and health care settings — within which they were able to explore aspects of their own thoughts and behaviors, as well as larger contextual factors. Moreover, they reported they had rarely if ever, examined these thoughts, perspectives, and contextual factors in past service encounters. We found the intervention components allowed for a deep exploration of personal perspectives on HIV management and its contextual influences, along with other concerns and challenges not directly related to participants’ decisions or abilities to engage along the HIV care continuum. This flexibility in intervention content (e.g., focusing on important non-HIV-related topics if the participant so desired) was seen as a vital aspect of the intervention components and of meeting participants’ needs. In fact, as noted above and as is common in an intervention trial, most participant in the present study did not achieve HIV viral suppression during the trial (40% achieved viral suppression). Nonetheless, rates of engagement in the components were high, among those who wished to prioritize HIV management, those who were not sure or were ambivalent, and also among those who were clear they did not. In the qualitative interview process, participants were asked to describe the ways in which they experienced each individual intervention component received. Participants commonly recalled the project activities in a holistic manner, and not always as distinct intervention components, as described in past research [2]. Nonetheless, in many cases, participants did remember both concrete details and tangible benefits of specific intervention components and/or noted the effects components had in their lives, which often included effects on HIV care engagement or HIV medication adherence as well as on other vital aspects of their lives. We found the underlying approach to each component grounded in the IIT-ICM (e.g., nonjudgmental, dignity-enhancing, autonomy-supportive, reflective of structural factors) was an important aspect of intervention acceptability and impact. We describe participants’ perspectives on and experiences with each intervention component in the sections that follow. We used gender-neutral pronouns (they/them/theirs) in the sections that follow because we did not know which pronoun series participants used to describe themselves. We also used pseudonyms and changed some identifying details to maintain participants’ confidentiality. As a reminder, the project’s field name was “Heart to Heart 2” and participants referred to the project using this name. In each section, we briefly describe the structure and content of the intervention component, followed by the results from the qualitative analysis that pertain to that component.

### Core intervention session (health education)

*Description*. The sequence of intervention components delivered to participants in each of the experimental conditions began with a core intervention session, a single, brief (< 60 min) individual session that had two main goals. The first goal was to provide or reinforce basic health education on HIV management considered necessary for engagement in other components (e.g., the expected frequency of HIV care visits and HIV medication adherence patterns). The second goal was to introduce the participant to the study ethos grounded in the IIT-ICM to thereby begin to foster a constructive relationship between the study and the participant to support future engagement in other intervention components. One example of how the IIT-ICM informed the core intervention session includes the assumption that not all AABL PLWH will want to attend or be able to attend HIV care appointments and/or that not all will take or be able to take HIV medication due to complex multi-level barriers, including structural barriers. This aligns with structural salience, since it locates the “problem” of poor engagement along the HIV care continuum largely at a structural, not individual, level of influence, and is thus a de-stigmatizing stance. Further, we took the stance that participants were experts on their own health, but that health education may be welcome, which aligned with a strengths-based and dignity-enhancing approach that is part of the IIT-ICM. These elements of the core intervention session were intended to build trust and foster future participation in components.

Derek was a cisgender, heterosexual Black man in their early 50’s, diagnosed with HIV 20 years ago, who came to the trial with a number of serious service needs, as well as previous largely negative experiences with health care settings. Derek described their first experience with the intervention components, namely, the core session, as follows:

This is really my first study that I’ve ever done. So, I have listened to rumors that you go in, you sit there for a couple of minutes, get your money and you’re gone. So, I didn’t really think that I was going to get anything positive out of this. But from the first visit [the core intervention session], I was impressed with the ambiance of the place, and it kind of made me feel comfortable enough to open up and start considering what I need to do to get my life together. […] I wasn’t used to being treated well at that point. So I came in, you guys treated me, welcomed me, very welcoming environment. And it made me feel comfortable, and made me feel, because I had been, my whole life had been living a lie up until then. So I didn’t feel like I had to lie, you guys weren’t judging me. And you really weren’t pushing me to do anything. You guys kind of wanted to know what I wanted to do with my life. So after the first visit I started really thinking about that. Like what can I get out of this, and how can I get this to help me move forward.

Derek’s experience, therefore, highlights the importance of introducing participants to the study ethos and stance as early in the trial as possible, to thereby foster engagement in the core session and future intervention components. In particular, Derek experienced the core session as non-judgmental, oriented around Derek’s own views on their health, and supportive of their autonomy (“you really weren’t pushing me to do anything” and “you guys kind of wanted to know what I wanted to do with my life.”) Overall, consistent with Derek’s experience, participants typically experienced the core intervention session, structured as a conversation and communicating aspects of the IIT-ICM, as a useful introduction to the spirit and ethos of the trial generally, including subsequent intervention components received. Importantly, health education was a useful vehicle through which to begin to establish this working alliance between the study and the participant and for the study to begin to earn trust and for the participant to begin to experience some level of trust. As noted above, these characteristics of the core session that participants found acceptable and engaging were described as generally lacking in typical health care settings.

### Component A: Motivational interviewing sessions

*Description*: This component was made up of four in-person, hour-long one-on-one sessions using the motivational interviewing approach, similar to the other components, as well as evidence-based motivational interviewing *techniques*. The goals of these motivational interviewing techniques were to foster motivation and readiness for behavior change by addressing individual and attitudinal barriers to HIV care continuum engagement, mainly health beliefs relevant to HIV care continuum engagement (e.g., outcome expectancies, self-efficacy, counter-narratives, the necessity of medication) and emotions (e.g., medical distrust, concerns/fears of HIV medication). In this component, interventionists maintained a non-judgmental stance and focused on uncovering and discussing relevant cultural and structural factors (e.g., past abuses of AABL populations in medical research and settings) that drive specific individual and attitudinal barriers to HIV care continuum engagement (e.g., medical distrust), and incorporating participants’ own views on how to best manage their HIV care and medication. Using one or two culturally and structurally salient video narrative segments per session to highlight key issues (e.g., distrust, fear, counter-narratives, poverty, stigma), interventionists used motivational interviewing-specific behavior change techniques including helping participants articulate their values, developing discrepancies between values and behavior, and identifying goals, barriers to achieving those goals, and the participant’s own solutions as they related to engagement along the HIV care continuum. Some examples of how the IIT-ICM informed this component include an explicit focus on specific barriers to HIV care continuum engagement that are common among AABL PLWH such as medical distrust, fear, and counter-narratives, which was intended to foster open communication and trust. One example of structural salience in this component was material grounded in an understanding of past and current systemic racism, which contributes to medical distrust, fear, and counter-narratives (sometimes called conspiracy theories). Counter-narratives about the origins and treatment of HIV were discussed and explored (e.g., that HIV was created to exterminate Black people), but not directly challenged. The videos were designed to signal to participants that it was acceptable to discuss concerns that are typically not encouraged in medical settings, such as medical distrust and counter-narratives if the participant so desired. Further, the component incorporated participants’ own views on how to best manage HIV and medication without judgment, a form of autonomy support.

A prominent theme found in the analysis was that participants who engaged in motivational interviewing sessions commonly reported that the sessions actively (and successfully) encouraged self-reflection and that the sessions were non-judgmental and non-coercive. In particular, participants noted the sessions provided them ample opportunities to identify, explore, and examine self-determined goals, and to address behaviors that participants themselves considered being in need of change, in contrast to those the research project staff, or a health care provider might identify as needing change. Moreover, participants stressed that at no point during their sessions did they feel stigmatized or pressured by project staff, regardless of the context. This, in turn, allowed participants to develop new skills, tools, and/or insights which played a role in their evaluating and making decisions about managing health and other aspects of their lives. Importantly, they highlighted that these types of experiences (self-determined goals, non-judgment) were largely absent in most other clinical and social service contexts in which they engaged.

Marcus was a Black, gay, cisgender man in their early 50’s, who was diagnosed with HIV as a teenager. At the time they enrolled in the study, Marcus was engaged in selling (or “diverting”) their HIV medication in part to provide funds to buy drugs. Their life was further complicated by legal problems since they had declined to report to their parole officer some months prior and thus were in violation of the terms of their parole, which triggered an arrest warrant that Marcus was actively avoiding. Despite these larger contextual challenges, including pressure placed on Marcus by a pharmacy that illegally sought to purchase their HIV medication bottles, Marcus was interested in evaluating the importance of HIV medication in their life. Marcus shared their experience:

The extreme honesty and their [the interventionist’s] ability to be 100 percent nonjudgmental [was important] because I can tell when somebody is being nonjudgmental because it's their job, but they don't wholeheartedly believe in what they're portraying. [...] There are times I even asked, "well, what is your opinion?" First response: "So what do you think it should be?" And that's good. […] It [motivational interviewing sessions] [nonjudgmentally] follows up on people and guides people -- and it doesn't really guide people; it guides people to make their own decisions and to comfortably make them, which is fine.

Marcus continued:

First of all, a person's only gonna change when they're ready to change. But do I think [the study interventionist] was instrumental in my becoming ready to make a change? I think that's a hundred percent fact, because regardless to whether or not individuals may show it at the time, conversations take place here. […] They're stuck in our mind. And they play out throughout the course of our travels when we run across an experience that we discussed here [in motivational interviewing sessions]. I guess the best way to describe is it puts arrows in our quiver in case we ever need them.

The IIT-ICM draws attention to structural and systemic barriers to HIV management. Marcus’s life was complex, in part related to structural and systemic factors, and they had a number of interrelated personal goals. Marcus’s quotes underscored the intention of motivational interviewing as an approach that supports autonomy in order to build durable intrinsic motivation for behavior change while guiding participants toward their personal health goals, which in this case included stopping crack use, trying hard not to sell their HIV medication, reporting to their parole officer (after a period of declining to report), and serving time in jail for the parole violation. Following Marcus’s lead about which goals to prioritize, rather than focusing on the trial’s primary outcome, namely, HIV viral suppression, was further consistent with the IIT-ICM and motivational interviewing approach. Notably, after serving time for the parole violation, Marcus re-started HIV medication.

Implicit in this set of findings is the importance of the individualized nature of the component; Marcus prioritized the parole violation and reducing substance use over HIV management at first, and motivational interviewing techniques could be applied to these goals. These findings also underscore the importance of harm reduction in the IIT-ICM. Marcus, like many participants in the study, was faced with any number of behaviors that could result in harm to themselves and others (e.g., not taking HIV medications, crack use, violating parole), and harm reduction may have played a role in fostering movement toward positive change in Marcus’s case. At the same time, forces in the larger environment such as pharmacies that illegally purchase HIV medication from PLWH remained a threat to Marcus’s decision to take HIV medication with high levels of adherence.

Cecil, who identified as a Black, cisgender, heterosexual man in their late 50’s and who was diagnosed with HIV nearly 30 years ago described their experience in this component:

I'm getting a better shot at looking at me. When I walk up into the door [at the project site] the mirrors come up and I get to see myself. Didn't actually like the person that I talked about. So I decided not to be that person anymore. […] Put it this way – I’m sitting up here talking, you’re my mirror. And sometimes, I might be missing something, and you might ask a question that I’d be missing, and I need to ask myself that question to help me get even a better focus on who I’m talking about. That’s me. […] Heart to Heart was my mirror. Heart to Heart was my mirror.

Cecil’s quote highlights the importance of self-reflection, which was certainly a critical aspect of the motivational interviewing approach used in this component. But motivational interviewing also seeks to strike a balance between fostering self-reflection and guiding participants toward change. And, for many, self-reflection was necessary, but not sufficient, for change. As Terrence, a Black, gay, cisgender man in their early 50’s who was diagnosed with HIV almost 20 years ago, described the importance of being guided toward change in the larger context of this component, including the need for autonomy support:

Again, I wasn't sharing my issues with anyone. So it was easier for me to walk around and tell myself that it was okay for me not to do those things because I didn't have anyone challenging me. So Heart to Heart kind of challenged me like that. So I needed to be challenged at that point because I was kind of on the fence on it [engaging in HIV care]. Like you know, I needed to get off the fence and put the footwork into rectifying the problem. Because it [not engaging in HIV care] was a problem.

Regarding this component, participants reflected on the importance of fostering or tapping into durable high-quality intrinsic motivation and self-reflection as mechanisms of behavior change in HIV decisions, and also stressed the importance of a culturally and structurally competent, non-judgmental, and pressure-free environment in guiding participants to and supporting these behavior change mechanisms.

### Component B: Pre-adherence skill building

*Description:* This component was designed to assist participants in building skills and durable habits to better manage adherence to HIV medication (and sustain high levels of adherence) or, for those choosing not to take HIV medication at the time, to better manage other health-related behaviors and/or to build skills for future HIV medication adherence. The six-week intervention component comprised two in-person sessions (at week 1 and week 6), and weekly brief telephone meetings with an interventionist during which participants identified and worked to resolve barriers to adherence within their physical and social environments. During the initial session, interventionists and participants assessed readiness for and identified barriers to adherence, established links between adherence and daily activities to build habits, put visual reminder aids in place (e.g., pill boxes, alarms), identified sources of long-term support and reinforcement, and established plans to minimize lapses in the event of missed doses. Habits were one means of addressing the cognitive biases that reduce HIV medication adherence to thereby improve adherence by making it automatic with less cognitive effort [56]. Weekly follow-up sessions provided an opportunity to explore barriers to and/or facilitators of habit formation and adherence skills. In the final session, any steps toward success and progress made were reviewed, and future plans for adherence supports were made. Participants were not always familiar with the concept of automatic habits or using reminders, and commonly appreciated the concepts. Examples of how the component aligns with the IIT-ICM include that the component reflected the major practical barriers AABL PLWH experience to viral suppression such as lack of privacy in home settings, which reflects structural salience, and the need to circumvent the emotions inherent in taking HIV medications through automatic and less effortful processes such as habits.

We found participants who attended the pre-adherence skill-building component discussed the degree to which introduction to the concept of habit formation, discussion of practical devices to manage adherence (such as pillboxes), and development of adherence-related skills played a role in their willingness and/or ability to become more highly adherent to HIV medication. Many discussed being provided with external memory aids such as daily or weekly pill boxes or alarms, building tangible skills which included developing habits and routines, and developing strategies to keep track of medications while away from home. For instance, many participants discussed the discovery of the importance of a reminder technique such as setting an alarm or having a visual reminder to take HIV medication or achieve other health goals (e.g., taking medication when one’s favorite television show started or after morning prayers). Indeed, these reminders, including aids such as pill boxes, and habits, were commonly lacking among participants in this study.

Emmanuel, who identified as a Latino, bisexual, and gender-fluid person in their early 40 s, who had been living with HIV for approximately 10 years, and who had struggled for most of those years with remaining in HIV care and adhering to HIV medication, explained:

[The interventionist] gave me a medical pillbox. She gave me the pillbox. Oh, yes. The pillbox is right on my table. The big blue one. I need the biggest one I can see. So I was so grateful and so appreciative that she gave it to me. That was when I got my medication, put it in the pillbox, and my alarm clock is right there. So when the alarm clock goes off, I get up, and I say, oh, it's time for me to take my medication. And I say my little prayers to God. I thank you for waking me up this morning. And I just take my medication.

Similarly, Derek, introduced above, added:

[The interventionist] helped me through that process too. She gave me a couple of pill [boxes]. One of them has a timer on it, so it goes off in the morning. So I’m very adherent to my medications now. And in terms of me again, I want to do it. She kind of really helped me to develop the skills that I needed to do that. Like I have a big pill cabinet in my bathroom now. So when I go in the bathroom in the morning, I can't forget to take my pills. There’s just like a big cabinet there. It’s just big. So it’s right there. So you kind of help me to develop the habit of making my pills visible for me so that I don’t forget. Because when I’m not feeling ill, it’s easy for me to keep my pills out of sight, out of mind. So that was a big issue for me. So now I’m very good in terms of that. I drive now also. So I have my pills in my car. I have them at home. Which is another thing that she told me, like keep your medication available wherever you are.

Participants also discussed a number of specific skills they implemented in their lives, particularly around habit formation, which they reported contributed to making their lives easier and that helped them to take their HIV medication more regularly, as well as attend to other health goals. TJ, a Black, cisgender, heterosexual man in their mid-50’s and diagnosed with HIV when they were in their mid-20’s, described the importance of their “command center:”

That's when she gave me the pill containers. […] She gave me two. I gave my mother one, because she really has an issue with taking her medication, and I just use the little round one with the seven days on it. I keep it at my bedside, which is my command center. So as soon as I wake up, I see them. Even if I don't take it right then, I get up and run in the shower and do whatever else I got to do. I normally go in there and take one before I leave the house, but if I do forget, once I get to the door it reminds me. So then I turn around – oops – and grab my pill bottle.

Relatedly, many participants expressed a sense of pride, accomplishment, and an improved belief in their ability to continue their newly formed HIV medication and other habits thanks to the skills built in this intervention component. Denise, a Black cisgender heterosexual woman in their mid-40’s, diagnosed with HIV over 10 years ago, described their experience:

[The interventionist] was just telling me how important it was to take my medicine. But that she wouldn't judge me. And that the only thing I feared [was] being judged. A couple times I became really saucy. And she just said, “I’m not judging you,” and [I] stopped. And that made me feel good. All of you all are real easy to talk to.

Thus, with respect to the pre-adherence skill-building component, participants recalled learning tangible, practical strategies for adherence in a nonjudgmental context, such as pillboxes, reminders, and habit formation, which they commonly found valuable for improving daily adherence practices. Further, these strategies were explored in the context of their living environments and social networks, and strengths were highlighted.

### Component C: Peer mentorship

*Description:* This component entailed peer mentoring provided by PLWH who were demographically similar and consistently engaged in HIV care with high medication adherence (that is, they were “successful” at HIV management). Peer mentors were long-term HIV survivors, as were most participants. Through informal counseling, peer mentors served as role models, providing practical tips and at the same time implicitly challenging negative social norms about the lack of engagement along the HIV care continuum among AABL PLWH. They were also an important source of social support, helping to combat social isolation, stigma, and other barriers to care and HIV medication. One pillar of the IIT-ICM is self-determination theory, which highlights autonomy, competence, and relatedness as fundamental human needs. Thus, one example of how the component aligns with the IIT-ICM includes the relationship between the peer mentor and participants, to foster relatedness. Further, peer mentors had credibility and could earn trust and reduce stigma. Last, peer mentors were typically culturally competent, and structural competence comes naturally to them given their lived experiences.

We found participants who received the peer mentorship component consistently reported experiencing the peer mentor as relatable and recalled what they described as a genuine peer-to-peer connection, underscoring the importance of relatedness in HIV management. For these participants, having peer mentors who had experienced or were experiencing similar struggles frequently facilitated open and honest communication and provided opportunities for developing more meaningful relationships between participants and an interventionist than were normally expected in a health care or social service setting. Roger, who identified as a heterosexual, cisgender, Black man in their mid-50’s, and who was diagnosed with HIV 15 years ago, explained:

You know. I call [my peer mentor] sometimes at night, you know when I’m struggling with stuff, you know what I’m saying? She guides me. You know like I was struggling about [whether to tell my mother I was living with HIV], and she was like tell her, tell her. I was like, I don’t know how to tell her [laughter]. She helped me get through that, you know?

Roger highlighted support they received from their peer mentor, and that “This place didn’t pressure me.” They contrasted the research project, however, with other settings, noting:

You know in prison the pressure was there. You know from the medical staff, you know? [They threaten] “I’m going to write you a shot [a punishment given to an inmate who violates a code or procedure] if you don’t take it [HIV medication].” Well, get to writing. You can’t force me to take nothing [laughter]. […] Oh yeah, we’re going to put you in seg [segregation]. Want me to write the paperwork for you [laughter]? How long I’m going to be there? I’ll go pack my stuff now. You can’t keep me there because I don’t want to take it [HIV medication].

Although Roger presented their experiences in a light-hearted manner, the coercive approach to HIV management commonly found in the prison setting was clearly detrimental to their health and well-being, since it resulted in Roger declining HIV medication while incarcerated. Indeed, as noted above, the IIT-ICM was designed to draw attention to contextual and structural factors that impede engagement along the HIV care continuum, including factors that deny dignity and restrict autonomy, as reflected in Roger’s quotes. These factors were elicited and discussed in intervention components, including this component.

Similarly, Monica, a heterosexual, cisgender Latina woman in their early 30’s who had been living with HIV for 10 years described their experiences with the peer mentor:

Well her [the peer mentor’s] past life and my life, it's similar. So maybe not everything, but there is a lot of things that we can interact and we come across. I can understand what she's saying. She can understand what I'm saying. I may not have been through everything she's been through but I've been through what I've been through.

Monica credited their peer mentor with playing a role in Monica’s decision to restart HIV medication and engage in a directly-observed-therapy program to support medication adherence.

Overall, participants commonly stressed that they felt truly cared for, listened to, and validated by peer mentors throughout the study, again often contrasting this experience in the research study with negative experiences in other social service or healthcare settings. Jared, a Black, heterosexual, cisgender man in their early 60 s, who was diagnosed with HIV at the age of 40 and who was struggling with medication adherence and substance use issues while in the trial, noted the level of care they experienced in this peer mentorship component:

Yeah, [my peer mentor was] real helpful. Hey, that's what I love about you. You check on people, that's good. It's not the point at when you're like on the parole office or anything – you're checking to see how I'm doing. Parole officer wants something you own.

Thus, Jared drew a contrast between being checked up on because one cares, in contrast to someone wanting something. Jared’s quote, similar to Roger’s above, further highlighted how commonly participants were located in contexts that restricted their autonomy and monitored their behavior, often with detrimental effects on health decisions and behaviors. Yet, a peer mentor could often successfully engage with participants and perhaps engender trust and communication through a deep, shared understanding of what it means to live with HIV. Participants reported that with this peer mentorship component, they were able to develop what they described as genuine, validating, and supportive relationships with individuals who shared similar backgrounds and experiences, challenges, and successes. This relationship, in turn, yielded benefits to HIV management and other areas of functioning in many cases.

### Component D: Focused support groups

*Description:* The focused support group component was designed to provide social, emotional, and instrumental support, reduce stigma, give acceptance or validation, and encouraged shifts in perspective to address barriers to HIV care engagement (e.g., regarding medical distrust and fear) and elicit and explore issues faced by PLWH, including those who use drugs, have mental health concerns, and/or are from sexual/gender minority status backgrounds. Six 90-min sessions were led by a clinician expert in group work and were convened every 2–3 weeks over a 4-month period. Topics for discussion were selected by participants, guided by a menu of options that included barriers to and decisions regarding HIV care/medication, reasons for not taking HIV medication, distrust and fear of and counter-narratives about HIV medication, race/ethnicity and social class and HIV medication, substance use and HIV medication, coping with HIV medication, managing pressure to take HIV medication in HIV care, mental health, stigma, managing communication with health care providers, and adherence to HIV medication. The menu of topics was intended to signal to participants that the focused support group component allowed for free and open discussion of aspects of HIV management that commonly affect AABL PLWH but that were typically challenging to discuss in typical health care and social service settings. However, the group was free to discuss any topic the members wished to focus on. Examples of how the component aligned with the IIT-ICM included that groups addressed major barriers AABL PLWH experience to engagement along the HIV care continuum that were not commonly discussed in other settings, which is de-stigmatizing and culturally and structurally salient.

We found social isolation was endemic in this sample, with deleterious effects on well-being and health. Participants who engaged in the focused support group component noted these group interactions provided a much-needed break from everyday routines often marked by loneliness, depression, and anxiety. As noted above, one goal of this component was to provide an opportunity for participants to discuss barriers to engagement along the HIV care continuum that were not commonly addressed in medical or social service settings, such as the decision to *not* take medication. Consistent with group theory, with respect to HIV management, peers generally had greater credibility than professional staff. Importantly, we found that participants developed social connections with individuals during the groups, some of which extended to life outside the group setting. Emmanuel, who was introduced above, described their support group experience:

The groups are fantastic. Yes. I met a lot of interesting people in the group. A lot of good brothers and sisters come to the group – different, diverse backgrounds; different people – and sharing and hearing their stories touched me. I even got emotional my last day, going to group. [...] Well, we talked about our past, our demons that some of us were facing. We talked about families in groups. We talked about goals in groups. And the main thing we talked about was taking our medication. That was the main thing: focus on taking your medication, because you're not going to be able to take that nice trip that you want if you're sick. To prevent you from getting sick, take your medication. Your medication is very important. Like I said, that made me change my whole thinking. Now I'm really [tuned in] with medication.

These groups were routinely described as a safe and confidential space within which to offer and receive emotional and practical support around HIV management issues or life in general. Thus, similar to other components, a flexible approach and the understanding that participants were whole people, not just PLWH, may have contributed to high levels of acceptability and feasibility for this component. Charles, a cisgender, Black, heterosexual man in their late 50’s who was diagnosed with HIV over 20 years ago, explained:

The group here was good. It really was. Yeah, because you could talk about anything. It don't have to be the medication. There are people there with different issues, and anytime I could talk about what's going on. It's helping, and it was good feedback from people who went through it. They would share their experience with it, do you know what I mean?

Likewise, Nelson, a heterosexual, cisgender Black man in their early 50’s living with HIV for almost 20 years described:

I think for the most part it's like when you hear like someone is going through a rough patch that you actually been through, and sometimes you think that you're not gonna get through it, but when hear, you know, the stories about, you know, this is where I was at and this is where I'm at now, it helps. It helps, because just knowing that it's possible or seeing that it's possible, is a big help. Just like -- just all types of different things, compliance with the meds. Like for example, I got like a nail infection, right, so they get real brittle. And the gentleman sitting next to me said, "Yeah, I had the same thing." So he was telling me that, you know, if you consistent with your meds and take what you doctor gave, it will go away. I've had this for a while now. I thought it was never gonna go away. [...] So, speaking of him, that was one of the things that, you know, if I wasn't in the group I wouldn't have known.

Social isolation and its sequelae were serious barriers to engagement along the HIV care continuum among participants. With respect to the focused support group component, guided conversations with peers sharing similar life experiences provided opportunities to candidly discuss struggles and successes. Moreover, this component was designed to elicit and attend to contextual and social factors common among AABL PLWH, and sometimes more common among AABL PLWH than their White peers, such as housing challenges, medical distrust, and counter-narratives. Thus, the focused support group component was described by participants as allowing for guided, yet exploratory, peer conversations that allowed for and encouraged participants to break from their daily routine and social isolation. They could discuss barriers and facilitators to HIV medication adherence and other shared goals, including aspects of HIV management that were not commonly discussed in typical care settings, such as medical distrust and counter-narratives. Further, at times participants developed longer-lasting social connections from the support group experience.

### Component E: Navigation

*Description:* Participants were randomly assigned to an experimental condition that included either a short (3-month) or long (6-month) version of navigation, which was designed to assist participants in identifying and overcoming structural barriers to effective HIV care and ancillary services. Individual, menu-based, highly focused sessions were held approximately weekly if needed, beginning with an in-person 30–60-min meeting during which participants’ barriers to HIV care/medication (including substance use and mental health issues) were reviewed, and Change Plan/Action Plans were created. Weekly (at minimum) check-ins via telephone, text message, email, and/or in-person meetings facilitated attendance, as needed, to menu items that included screening and “Fast Track” referrals for housing, substance use, mental health, and other concerns; joint problem-solving barriers to appointments; and providing accompaniment to health care appointments as needed. Examples of how the component aligned with the IIT-ICM include that it was designed to address the primary structural barriers that AABL PLWH experience to HIV care, HIV medication, and ancillary services such as substance use and mental health. Because it attended to structural factors that impede engagement along the HIV care continuum, including those driven by systemic racism and structural inequality, it was structurally salient.

As is noted above, many participants recalled frequently having to navigate complex and often frustrating or even dehumanizing bureaucracies, which they described as a common challenge related to HIV medication adherence. For instance, participants often discussed the intricacies involved in finding services and staying connected to care both directly and indirectly related to HIV medication. James, a Black, cisgender, gay man in their mid-30’s who had been living with HIV for about six years described their experience accessing suboxone for pain management:

Yeah, but you know literally like [my navigator] helped me like really literally bridge the gap so I can actually get the [pain medication], because the thing about it was at [health care setting] and they were administering the suboxone. I couldn’t take any home. So she helped open the door for me to get the pain management. And I actually get the scripts now and they deliver it to me.

Participants struggled most often with housing and accessing primary care, mental health services, and substance use support. These struggles and hassles were reported to negatively affect their sense of dignity and self-worth. In their interactions with navigators, however, participants discussed developing a sense of their own value, which helped them feel enabled to directly ask for what they needed from their providers. Derek, introduced above, also described prior negative previous experiences with health care:

But again, my belief system. I didn’t believe that people were going to help me because I had been nonadherent [to HIV medication]. I had been chemically addicted and running around. So coming out of all of that stuff really put me in a bad place mentally. And I didn’t believe that I could do some things. And the people here at Heart to Heart kind of believed in me. They believed in me for me. And once I started the process everyday became a little easier. When I went to change the primary care doctor. I did not think that, because I have never really had a good primary care experience. It’s always been you’ve got to wait. And then when you get in the doctor’s rushing you out. They don’t have time to discuss issues with you. So I didn’t think I was going to be able to find a good primary care doctor. I have had a number of them. So now that the primary care doctor that I have now, every time I go, I’m more surprised at the reception that I receive from them. If I have a question, they have a lot of services. Like they have social services on the floor. They have case workers on the floor. So if I need something that person is usually available on the floor and they’ll direct me right to them. So I feel like my needs are important to them. And that’s something I never gotten at a primary care facility.

Similarly, Angel, a Latino cisgender, bisexual man in their early 30’s diagnosed with HIV as a teenager, noted:

But the navigation part, [the navigator] was really good at navigating stuff. It was almost like I needed her, but I did it all on my own anyway. It was like a boost with her. It was like a boost. No, I did everything on my own. I had found a new apartment and everything.

The intervention components were individualized, as noted above, and interventionists were able to respond to the primary problems that participants wished to address, whether related to HIV or not, including in the navigation component which focused on structural barriers to health. Many participants also reported that they felt able to discuss substance use and other potentially sensitive and/or stigmatizing issues in a safe, supportive environment, and frequently noted that staff was able to “meet them where they are at.” Derek, introduced above, described:

I really didn’t think that I was going to be successful at any of these things when I got to Heart to Heart. People told me I could get another [health care provider], because I had given up by the time I got here. You guys told me that I could [be] back in [substance use] treatment. That I can find treatment that would be suitable for me. That I could get back on the medication, that I could become adherent. That I could achieve undetectable viral load. Which all of those things came true for me. Another one, I did not think that I was going to be capable of becoming employed because I had been out of work for a number of years. And you guys encouraged me and told me to just put the resume out there, see what happens. Don’t project what’s going to happen just do the foot work. And so I was able to do that. And one of the first jobs I applied for called me. And I applied for a job as a line worker, and when they looked over my resume, they felt that my experience dictated that I could supervise on the site. So I came in as a supervisor. I’m supervising now. Really beyond my wildest dreams. So those are just a few of the things that have happened to me.

Thus, Derek experienced a cascade of changes and benefits from their work in the navigation component. Primary among these was linkage to suitable substance use treatment, which contributed to their re-initiating HIV medication, which, in turn, allowed Derek to rejoin the workforce at a supervisory level.

The receipt of material and practical support from the project was a prominent theme in participants’ descriptions of components, likely related to the fact that all participants received the navigation component for either three or six months. Regarding this component, participants noted that the project staff was, at times, able to assist them with locating new healthcare providers and mental health services, navigate frustrating and often unsympathetic or even hostile bureaucracies, obtain basic needs from food pantries and other community-based organizations, and provide support and guidance to access housing. As noted above, these aspects of the navigation component were intended to address social inequities and circumvent structural barriers to HIV management. Yet a lack of high-quality housing placements, and the generally poor availability of mental health care and substance use treatment services commonly impeded successful navigation efforts. In many cases, participants’ needs went unaddressed, despite the navigator’s best efforts.

## Participant Recommendations for Improvement

Although participants reported overall satisfaction with the study’s individual intervention components, as well as with the research program as a whole, there were nonetheless a number of gaps identified. By far the most frequently mentioned area where improvement was needed was related to the length of the intervention components and intervention period. For many participants, their time in the intervention components and intervention period was far too short, and as a result, participants frequently reported feeling unexpectedly disappointed when their time in the project ended, even after recalling that they were made aware of the duration of their study participation in advance. Noting that they would have liked more time for support and services, for instance, Jordan, a cisgender heterosexual Black man in their 60 s and diagnosed with HIV in their late 30’s, noted the following:

Yeah, they should let it go on like three or four years. [...] Well no, because like I said, I liked the program and stuff. I wouldn't change none of that. I just would add on some more extended time.

Similarly, Roger (introduced above) suggested the following:

Just some of the big components are a little too short. And they can dig a little deeper, you know? [...] Different ways to help people take their medicine.

In addition to extending the length of the intervention period and intervention components, several participants not assigned to focused support groups stressed the need for more group interaction.

### Intervention component acceptability (quantitative measure)

Acceptability of intervention components was assessed quantitatively by examining proportions who attended each component and rated it as ‘quite a bit’ to ‘very’ helpful (Table 3). For all components, proportions of attendees endorsing these levels of helpfulness were satisfactory, with approximately 70% or greater rating the component as ‘quite a bit’ to ‘very’ helpful.

**Table 3:** Acceptability of intervention components at the final follow-up assessment (among those who attended the component)

**Component**	**% (N) reporting the component was quite a bit to very helpful**
Component A: Motivational interviewing sessions	76.4 (146/191)
Component B: Pre-adherence skill building	68.3 (114/167)
Component C: Peer mentorship	77.8 (133/171)
Component D: Focused support groups	78.9 (138/175)
Component E: Navigation – short version	74.4 (145/195)
Component E: Navigation – long version	74.0 (148/200)

### Intervention component feasibility (quantitative measure)

Feasibility was assessed quantitatively by evaluating proportions of participants attending intervention components to which they were assigned, and the extent to which participants remained engaged in the component, as appropriate (Table 4). Each component was attended at least once by 80% or more of assigned participants; in most cases, this proportion exceeded 90%. Navigation (both short and long versions) had the highest level of attendance, with > 95% of assigned participants attending at least one in-person meeting. Motivational interviewing sessions and peer mentorship also had high attendance levels, with > 90% of assigned participants attending at least one session. Pre-adherence skill building and focused support groups were each attended at least once by 80% of assigned participants. Thus, intervention component feasibility was high.

**Table 4:** Feasibility: Attendance at assigned intervention components (*N* = 512)

**Component**	**% attended or M(SD), (N attended/N assigned)**
**Core intervention session**	
Attended	97.9 (501/512)
**A. Motivational Interviewing Sessions**	
Attended at least one session	95.0 (228/240)
**B. Pre-adherence skill building**	
Attended at least one meeting	79.5 (194/244)
**C. Peer mentorship**	
Attended at least one meeting	90.6 (211/233)
**D. Focused support groups**	
Attended at least one group	79.5 (194/244)
Attended all six groups	51.2 (125/244)
Total groups attended	3.89 (2.49)
**E. Navigation—long**	
At least one in-person contact	96.5 (249/258)
Total contacts (all modes)	6.10 (3.31)
**E. Navigation—short**	
At least one in-person contact	96.9 (246/254)
Total contacts (all modes)	2.74 (2.76)

## Discussion

The persistent and serious racial/ethnic inequities in engagement along the HIV care continuum, along with the growing population of long-term HIV survivors, signal the need for enhancements to conceptual approaches, behavioral interventions, intervention development methods, and health care delivery models. The population we focus on in the present study is AABL PLWH who are mainly long-term HIV survivors and who are poorly engaged along the HIV care continuum, primarily as a result of serious structural and social-level barriers such as extreme poverty, unstable housing, and challenges accessing high-quality care and services. Further, the barriers that AABL PLWH experience to the HIV care continuum also impede engagement in HIV research. In response to this need for new models and interventions for this subpopulation of AABL PLWH, in past research, we developed a conceptual model that integrated critical race theory, the harm reduction approach, and self-determination theory, called the IIT-ICM. This model then guided the development of a set of six behavioral intervention components designed to be culturally and structurally salient and address the primary barriers that AABL PLWH experience to HIV care continuum engagement, tested in an optimization trial [2]. The present qualitative and exploratory study uncovers and describes participants’ views on these specific behavioral intervention components in more detail. We explored the interventions components’ acceptability, feasibility, mechanisms of action (whether related to the IIT-ICM or otherwise), ways the component may have influenced the participants’ thinking or behavior, and ways components could be improved.

As noted above, acceptability and feasibility are the foundations of engagement with an intervention, defined as the depth of involvement with the behavior change process [17, 18]. Interventions and intervention components are highly unlikely to be effective if participants do not find them useful and/or do not attend intervention activities at high rates. We found in qualitative and quantitative results that the intervention components explored in the present study were both highly acceptable and feasible, and in qualitative results, participants also reported a range of positive social, emotional, and behavioral effects as a result of their engagement in the components. We interpret these high rates of acceptability and feasibility, engagement, and reports of effects as related in part to the core elements of the IIT-ICM, along with the specific technical aspects of the component. For example, the IIT-ICM locates the “problem” of poor engagement along the HIV care continuum as largely related to structural-level factors, but not primarily a failing of individual AABL PLWH. In this context of understanding and acknowledging structural barriers, participants are provided with individualized services that take an autonomy-supportive approach. The IIT-ICM also highlights the importance of guiding participants toward positive change using evidence-based techniques; it is not a passive approach. Although the primary outcome of the optimization trial was HIV viral suppression, and the intervention components were designed with this outcome in mind, participants generally experienced benefits from the intervention components whether or not they valued HIV viral suppression or were taking HIV medication at the time they engaged in the trial. In the sections that follow we discuss findings as they pertain to each of the intervention components.

There is utility in exploring participants’ perspectives on how a component operates in qualitative research. One way to understand how participants experience the intervention components and their mechanisms of action are to highlight participants’ explicit and implicit views on qualities found in the intervention components that they experience as insufficient or lacking in other health care and social service settings. We found that the core intervention session that all participants received played an important role in interesting participants in the study by orienting them to the study’s overall ethos (“I was impressed with the ambiance of the place, and it kind of made me feel comfortable enough to open up and start considering what I need to do to get my life together”). The core intervention session served a trust-building function, and participants described experiencing trust and viewing staff as trustworthy as largely absent in other types of settings.

The core session was followed by the navigation component as means of addressing structural barriers to HIV care settings and ancillary services. Indeed, participants cannot take HIV medication until they are linked to a healthcare setting, and in many cases, mental health and substance use concerns warrant attention before prioritizing HIV care. In fact, participants generally required assistance with housing and accessing primary care, mental health services, and substance use treatment. But the extent to which health care and social service settings were experienced as frustrating or even dehumanizing bureaucracies cannot be over-stated. Participants reported experiencing poor treatment and stigma in HIV care settings because of their not taking HIV medication and related to their substance use. The navigation component certainly could not eliminate all of these types of barriers to engagement in HIV care. However, navigation was experienced as a needed and supportive service, sometimes to solve concrete challenges that participants faced, and other times to provide encouragement in the face of serious obstacles, or both. Participants reported that navigation was useful for addressing a range of concerns outside of HIV management, such as pain management and obtaining a job. Results suggest that the emotionally supportive and encouraging aspect of navigation was as important as the concrete services provided. Yet insufficient housing placements, numbers of mental health care practitioners, and a lack of substance use treatment settings meant that participants’ needs often went unaddressed.

With respect to the motivational interviewing session component, participants highlighted the importance of developing self-directed, high-quality, durable intrinsic motivation for behavior change, supported in large measure by motivational interviewing techniques (“When I walk up into the door the mirrors come up and I get to see myself,” “it guides people to make their own decisions”) and the general motivational interviewing approach, in an environment of non-judgment. Participants commonly emphasized that their personal health decisions were not made by the study staff, but by the participant themselves. The study staff were seen as playing a role in health decisions, but participants saw themselves as the ultimate experts on their own health behavior. The salience of this particular theme, repeated by many participants, suggests that personal autonomy over health may not always be respected in health care settings. Further, the individualized approach used in the component to identify personal goals was salient, particularly given the complexity of participants’ lives. The importance of harm reduction, one of the three pillars of the IIT-ICM, applied to any number of challenges and health behaviors (e.g., parole violations, selling HIV medication, substance use) was apparent. Implicit in these findings is the significance of structural and cultural competence on the part of intervention staff [25], who were trained in the structural/cultural factors that typically impede engagement along the HIV care continuum [57]. In contrast, if the IIT-ICM and this component had lacked a focus on these larger structural and cultural factors, the acceptability, feasibility, and impact of the component may have been diminished. The importance of structural and cultural salience of components, and structural and cultural competency of the interventionists, is relevant to the other components as well.

The IIT-ICM draws attention to the importance of relatedness, one aspect of self-determination theory. We found the peer mentorship component fostered meaningful relationships between participants and the peer mentor, which in turn allowed the peer to serve as a role model for HIV management, and to provide guidance and support. Trust is a crucial aspect of clinical and professional social supportive interactions, and peer mentors may be well-positioned to develop trusting relationships and open communication, while serving as role models. As AABL PLWH themselves, and also long-term HIV survivors, peer mentors bring high levels of structural and cultural competence to the role, along with expertise in many of the contextual factors that influence participants on the course of HIV management such as substance use challenges, trauma, and incarceration. In the present study, similar to other studies that include peers as interventionists, peer mentors must disclose their HIV status to others on a regular basis, and grapple with the constellation of serious risk factors that AABL PLWH experience to engagement along the HIV care continuum, and that they themselves may have faced, or may be currently facing. Peers who serve in service and clinical roles may require high levels of supervision and support because they do not always have the educational experiences comparable to their professional cohorts and because serving as a peer requires drawing from and often disclosing their own life experiences to build close relationships with participants or clients. Thus, findings suggest peers may be at risk for emotional exhaustion and burnout [58], and can find the work with PLWH participants “triggering” with respect to their own traumas, losses, mental health concerns, and substance use challenges. Moreover, participants noted they contacted their peer mentors outside work hours, suggesting the need to support peer mentors in setting boundaries. At the same time, serving as a peer mentor can have positive effects on the peers themselves, as well as on their participants [58]. Thus, the present study highlighted high levels of acceptability and feasibility of a peer mentor intervention component and also suggests the need for greater attention to peer mentor selection, training, and support. In future research, we will explore the effect on peers of serving in the role of a peer mentor.

The primary mechanism of action for the focused support group component, in theory, was facilitated peer-to-peer interaction to increase social support and reduce stigma. Findings suggested that the component was successful in reducing experiences of social isolation and that peers served as credible sources of information about HIV management and coping with challenges generally. Guided by the IIT-ICM, the group facilitator was primed to attend to and allow exploration of contextual factors that influence HIV management or factors that are indirectly related to HIV management. Participants noted this flexibility (“you could talk about anything. It don't have to be the medication”) and this may have contributed to high levels of acceptability.

The pre-adherence skill-building component was the last component delivered to participants and showed satisfactory levels of acceptability and feasibility. The need for and utility of memory aids such as alarms and reminders, and pill boxes, are well established in the field [59]. Yet, participants found these aspects of the component to be novel and also helpful. This finding may reflect challenges inherent in long-term HIV survivorship. Although AABL PLWH are generally offered adherence support and training early in their HIV trajectories in health care and social service settings, the present study suggests adherence support and practical tools such as pill boxes may be better conceptualized as an ongoing need. Further, this population of AABL PLWH may not have the financial means to purchase pill boxes, but these can be routinely provided in health care and social service settings. Findings also suggest that the concept of habit formation was new to participants and interesting to them, as well as useful in many cases. There can be stigma associated with poor HIV medication adherence (“the only thing I feared [was] being judged”). Thus, a nonjudgmental approach was critical to the pre-adherence skill-building component, as we found with other components, as it allowed participants to discuss HIV medication non-adherence. Structural and cultural competence of interventionists are prerequisites for carrying out this component; for example, housing arrangements may preclude privacy for medication storage and taking, and extreme poverty means participants may not have been able to purchase pill boxes. Further, an individualized approach to intervention delivery is necessary, since not all AABL PLWH wish to take HIV medication at any given time.

Rates of attendance were acceptable for the pre-adherence skill-building and focused support group components (Table 3), but notably lower than for the other components. We attribute this mainly to aspects of the optimization trial, with specific clinical issues impeding attendance as secondary. As noted above, the number of components provided to participants varied across the 16 experimental conditions, and some experimental conditions provided more components than others. In past exploratory research, we did not find evidence that attendance in some components reduced attendance in other components. In other words, these relatively lower attendance rates do not appear to be a function of which experimental condition participants were assigned to (data not shown). Pre-adherence skill building was the last component offered, and in some cases, participants were unable to attend, declined to attend (perhaps because they did not wish to discuss taking HIV medication), or we were unable to locate them at that time. Groups could not be scheduled at a time that was convenient for all participants, in contrast to other components, which may have reduced attendance somewhat. Further, some participants reported negative past experiences in groups in other settings (such as in substance use treatment) and declined to attend [60]. The COVID-19 pandemic required that we switch abruptly to a virtual format mid-way through the study [39, 61], which reduced attendance in the focused support group component somewhat. Participants in general did not have the smartphone or computer equipment or technical capabilities to use a Voice over Internet Protocol for virtual group activities [39]. The other components could be provided over the phone. Regarding acceptability, these two components were found acceptable, but the acceptability of pre-adherence skill building was somewhat lower than the other components. We attribute this to the fact that this component focused on building adherence skills and habits, but not all participants were taking HIV medication or seeking to achieve HIV viral suppression during the trial. Thus, that component may not have been useful to them at that time. Overall, the study results suggest that all six intervention components are acceptable and feasible for this population, despite minor variations as shown in Tables 3 and 4.

Adverse childhood experiences were prevalent in this sample. Half the sample reported four or more such experiences, the commonly accepted cut-off for putting individuals at high risk for toxic stress physiology and later adverse physical and mental health outcomes, including depression [62]. In the United States general population, 16% evidence four or more types of adverse childhood experiences [62], highlighting the elevated prevalence of early risks this population faces. Adverse childhood experiences typically affect social development as well and can contribute to later social isolation [62]. The intervention components were not designed to address the downstream effects of adverse childhood experiences in this population, but have potential to reduce or eliminate them. Certainly, the optimal means of addressing the effects of adverse childhood experiences in this population warrants future study.

The present study extends the knowledge base on behavioral intervention components for AABL PLWH. Conceptualizing interventions as being comprised of separate components that can be tested individually is a relatively new area of research. This subpopulation of AABL PLWH experiences chronic severe poverty, unstable housing, substandard cell phone access with limited “minutes,” poor health, unemployment, a general lack of structure to one’s days, and substance use concerns, as described in the present study. The high levels of engagement in the intervention components underscore the acceptability of intervention components as well as the expertise of the research team in managing longitudinal research studies with high levels of retention [11, 63], and the fact that research studies may be better resourced than some social service settings. We found that at least some participants would have appreciated a longer duration of components, and those who did not receive groups highlighted their preference for group-based activities, findings that will inform future studies. Future research will also examine ways to make the future multi-component intervention sustainable once the research component ends, drawing on ideas such as creating and supporting a “Heart to Heart alumni network,” training participants to become peer educators and support group leaders, and implementing the new intervention (to be determined) in community-based and health care settings.

Systemic racism is a form of racism embedded in the laws and regulations of a society or an organization and that manifests as discrimination in areas such as criminal justice, employment, housing, and health care [24]. Findings in the present study underscore the pervasiveness of systemic racism in the lives of AABL PLWH and its deleterious effects on quality of life, well-being, and HIV management. Challenges accessing high-quality HIV and ancillary care (e.g., pain management) can be interpreted in this context, along with other risk factors common in this study such as poor quality or unstable housing, low rates of employment, and high rates of involvement in the criminal justice system. The IIT-ICM is informed by the influence of systemic racism in the larger context in which AABL PLWH are located and components were intended to counteract its effects. The present study did not include an explicit assessment of structural and individual-level racism and discrimination and its effects on AABL PLWH, gaps we will attend to in future research.

### Limitations

The study has limitations including the possibility of social desirability bias. We sought to minimize this potential bias during the interview process by asking general questions before specific questions and reminding participants they could and should feel free to decline to answer any question without penalty. The analyses did not yield findings about any domains not included in the IIT-ICM, but these certainly play a role in HIV management for AABL PLWH. We will attend to emergent themes as we continue to study and refine the IIT-ICM and understand its application to intervention development. The sample was comprised of mainly African American/Black cisgender men, which limited our abilities to explore sex, gender, sexual orientation, and racial/ethnic differences. Moreover, we did not carry out qualitative interviews in Spanish, because the number of monolingual Spanish-speaking participants in the optimization trial was very small. Yet, this is a limitation of the present study. As a qualitative and exploratory study, we do not examine the efficacy of the intervention components or identify the optimal combination of components. These will be presented in future research and can serve as a type of triangulation of qualitative results presented here [64]. Nonetheless, the present study advances our understanding of intervention component development for AABL PLWH, independent of the optimization trial’s outcomes.

### Implications for health care settings and future research

Study findings suggest serious gaps in the set of medical and social services available to this subpopulation of AABL PLWH, including those who are long-term HIV survivors. Past research has highlighted that HIV viral suppression, even once achieved, is not commonly sustained in challenging contexts such as those in which AABL PLWH are embedded [8, 9]. The present study supports the utility of intervention components with a structurally and culturally salient focus and grounded in the IIT-ICM. Salient characteristics of the IIT-ICM are described by participants as largely lacking in most health care and social service settings, which appears to impede AABL PLWH’s engagement in these other settings. Results from the present study will be used, along with other analyses carried out as part of this optimization trial, as part of a future decision-making process led by the research team to determine the next steps in this program of research, which may include a randomized controlled trial of a combination of the components or another optimization trial. Increasing sustained rates of HIV viral suppression in this population of AABL PLWH with serious barriers to engagement along the HIV care continuum is clearly challenging, as the present paper describes.

## Conclusion

The behavioral intervention components described in this study have high acceptability and feasibility among this sample of AABL PLWH. Further, a wide range of positive effects were found related to HIV management and other aspects of their lives. The IIT-ICM is a flexible model for intervention development that can be applied to other types of health inequities or health disparities and warrants further investigation. These study findings will be of interest to intervention developers generally and those interested in the MOST framework, along with the public health community committed to reducing racial/ethnic inequities in HIV and ending the HIV epidemic.

## Supplementary Information

**Additional file 1. **Heart to Heart 2 (HTH2) semi-structured interview guide.

## Data Availability

Data are available upon reasonable request from the corresponding author.

## Abbreviations

AABL: African American/Black and Latino
HIV: Human immunodeficiency virus
IIT-ICM: Intervention Innovations Team integrated conceptual model
MOST: Multiphase optimization strategy
PLWH: Persons living with HIV
PTSD: Post-traumatic stress disorder
